# Longitudinal trajectories of mental health in Australian children aged 4-5 to 14-15 years

**DOI:** 10.1371/journal.pone.0187974

**Published:** 2017-11-13

**Authors:** Daniel Christensen, Michael T. Fahey, Rebecca Giallo, Kirsten J. Hancock

**Affiliations:** 1 Telethon Kids Institute, Subiaco, Western Australia, Australia; 2 Department of Statistics, Epidemiology and Data Science, Swinburne University of Technology, Victoria, Australia; 3 Murdoch Childrens Research Institute, Victoria, Australia; 4 Department of Paediatrics, The University of Melbourne, Victoria, Australia; Pennsylvania State University, UNITED STATES

## Abstract

Mental health can affect young people’s sense of wellbeing and life satisfaction, their ability to participate in employment and education, and their onward opportunities in life. This paper offers a rare opportunity to longitudinally examine mental health in a population-representative study of children aged 4–5 years to 14–15 years. Using data from the Longitudinal Study of Australian Children (LSAC), this study examined maternally-reported child mental health over a 10 year period, in order to understand their initial mental health status early in life and its change over time, as measured by the Strengths and Difficulties Questionnaire. Longitudinal models were fitted from ages 4–5 to 14–15 years. Results showed that child sex, maternal mental health, socio-economic status (family income, maternal education, neighbourhood disadvantage), maternal hostility, and child temperament (persistence, sociability, reactivity) are all independent contributors to child mental health at age 4. These effects largely persist over time, with the effects of maternal mental health increasing slightly over time. Persistence of these effects suggests the need for early intervention and supports. The independent contribution of these factors to child mental health suggests that multi-faceted approaches to child and maternal mental health are needed.

## Introduction

Mental health is strongly associated with young people’s sense of wellbeing and life satisfaction, their ability to participate in employment and education, and their onward opportunities in life. In high income countries it is estimated that 10–20% of children and adolescence experience internalising (i.e., anxiety and depression) and externalising problems (i.e., aggression and oppositional behaviour) [1–4]. More recently, it was estimated that almost one in seven Australian children aged 4–17 years had a mental disorder in the previous 12 months, equivalent to 560,000 children nationally [1]. Mental illness is a leading cause of health-related burden worldwide among young people (aged 10–24 years) [5], and has been identified as a cause of disability adjusted life-years lost in children and young people in Australia [6]. The short- and long-term consequences of mental illness can be profound, affecting educational attainment, substance abuse, violence, reproductive and sexual health, employment, relationship difficulties and suicide [7], each of which can then affect the mental wellbeing of the next generation.

Many studies have identified risk and protective factors associated with children’s mental health difficulties [5, 7–12]. For instance, data drawn from 4,735 children participating in the Longitudinal study of Australian Children [13], found that cultural and linguistic diversity, Indigenous status, gender, maternal age, maternal education, family income, parenting style and neighbourhood disadvantage were associated with children’s emotional and behavioural difficulties as measured by the Strengths and Difficulties Questionnaire (SDQ) at 4–5 years.

Whilst this body of work has generated important evidence about what increases children’s risk of mental health difficulties, a key methodological issue is the almost exclusive reliance on cross-sectional designs. Although the early origins of mental health difficulties are thought to begin in childhood [14–20], there are remarkably few longitudinal studies that have examined the course of mental health difficulties from childhood into adolescence. Longitudinal analyses using repeated measures offer a richer understanding of the natural history or pattern of mental health symptoms for children as they make the transition into adolescence. In addition to assessing stability and change, there is an opportunity to identify factors that are associated with early mental health and the further development of mental health difficulties over time. Importantly, this work can identify risk factors associated with enduring adverse consequences for children’s mental health, generating evidence to inform approaches to the prevention, early identification and mental health support for children at risk of long-term mental health difficulties.

Only a few studies have examined children’s mental health across multiple time points and identified factors associated with change and/or persistence. In a nationally representative study of 13,568 adolescents in the United States, 9%reported moderate to severe depressive symptoms, and factors associated with persistence of symptoms one year later included being female, having fair or poor general health, school suspension, poor family relationships, and health care utilisation [21]. In another study of 2,587 British children (aged 5–15 years), mental health was assessed at two time points over a three year period using the SDQ [22]. A high degree of stability in mental health over time was observed with externalising behaviour difficulties (compared to emotional problems), having reading difficulties, physical health problems, living in a single-parent or step-parented family, being exposed to parental separation and parental mental illness, and the loss of an important friendship.

At three time points over a 6-year period, a recent study assessed emotional behavioural difficulties of 630 children and adolescents (aged 6–18 years) in Germany using the SDQ [23]. The vast majority of children had scores in the ‘normal’ range across the 6 years, with 1–16% moving from normal to abnormal over the period. A small proportion (2–3%) had persistent problems in the ‘clinical’ range across the 6 years. The factors associated with emotional behavioural difficulties at the 6-year follow-up were SDQ scores in the clinical range at the start of the study, low socioeconomic status, and being male.

Taken together, these studies highlight the value in assessing children’s mental health over time, and to identify the factors that predict later outcomes. Few studies, however, have examined changes in mental health across the transition from childhood to adolescence using more than two or three time points. The transition from childhood to adolescence is a critical period of development. It is a time when young people are at increased risk of self-harm and suicide. For instance, approximately one in ten Australian 12–17 year olds reported having ever self-harmed [1]. The risk of self-harm and suicide is higher for children with mental health issues. Zubrick et al [24] found that 18% of all 12- to 17-year old young people with any mental health disorder said that they had engaged in self-harm in the past 12 months, with almost half of all young people who met criteria for major depressive disorder reported that they had engaged in self-harm in the past 12 months. Given these risks for young people, there is a need to better understand the ‘normal’ trajectory of mental health symptoms for children in the general population, and to identify the early life factors that may place some children at risk of poor mental health as they make the transition into adolescence.

We had a unique opportunity to address this by examining the stability and change in maternally-reported child mental health over a 10-year period for over 3,000 children participating in the nationally representative Growing up in Australia: Longitudinal Study of Australian Children. Children’s mental health was assessed biennially using the SDQ at 6 time points from aged 4–5 years to 14–15 years. We also sought to explore the relationships between key social risk factors and change in children’s mental health over time. Drawing upon a biopsychosocial model of child development that focus on the interplay of genetic, psychosocial and broad social or structural factors in determining health outcomes for children [25, 26], we were particularly interested in investigating child temperament, parent mental, parenting behaviour and family socio-economic status as factors associated with changes in children’s mental health over time. Each of these factors is discussed below.

Temperament has been shown to be an important antecedent of childhood emotional and behavioural problems. Temperament can be defined as “constitutionally based individual differences in behavioural style, relating to affect, activity and attention that are visible from early childhood” ([27] p.228). In a study of 1,037 children from the Dunedin Multidisciplinary Health and Development Study, Caspi found that an “undercontrolled” temperament at age 3 years predicted externalising and internalising problems during adolescence and that an inhibited temperament during childhood predicted internalising problems during adolescence [28]. The Australian Temperament Project, a community based longitudinal study which has followed a large community sample from infancy to adulthood, has shown that temperamental dimensions including reactivity (volatility and negative emotionality) and inhibition or shyness (low approach) are associated with a higher levels of externalising and internalising problems respectively throughout childhood and adolescence, and lower levels of social competence [29]. Seeking to extend this work, we aimed to explore the relationships between the three dimensions of temperament (persistence, sociability and reactivity) and change in children’s mental health over time. We hypothesised that low persistence, high reactivity, and low sociability would be associated with higher SDQ scores (or lower social-emotional wellbeing) at age 4–5 years.

Another well-established risk factor for child mental health problems is parent mental health [7, 30–32]. There is strong evidence for the adverse effects of maternal depressive symptoms during pregnancy and the first year postpartum, with systematic reviews reporting increased risks of internalising and externalising behaviour problems from infancy to adolescence [33, 34]. A recent population-based study of over 1,000 Australian women revealed that approximately 9% of women report persistently high clinically significant depressive symptoms from pregnancy to 4-years postpartum, and a further 30% report subclinical levels of symptoms over time [35]. Children of both groups of mothers were at least two times more likely to have emotional–behavioural difficulties in the clinical range on the SDQ at age 4 years than children of mothers reporting minimal symptoms. Similarly, in a register study of all Danish children born between 1980 and 1994, parental mental health difficulties were found to be positively associated with virtually all offspring psychiatric outcomes [36]. Whilst parent mental health is clearly associated with child outcomes, it is important to note that studies to date have examined the associations between parent mental health difficulties and child outcomes measured a single time point. To the best of our knowledge, no studies have examined children’s trajectories of mental health over time according to their exposure to maternal mental health difficulties. This will provide important prognostic information about how children of parents experiencing mental health problems fare over time as they become adolescents.

The adverse effect of parent mental health difficulties on parent-child interactions has been identified as one mechanism by which parental mental health impacts on children’s emotional and behavioural development [37]. Two critically important and inter-related aspects of how parents interact with their children are their levels of hostility (i.e., anger, yelling, rejection of child), and the degree of warmth (i.e., displays of affection, closeness, acceptance, responsiveness) in their relationship with their child [38]. In particular, hostile parent-child interactions have been associated with emotional and behaviour difficulties among children and adolescents [39–42], and low maternal warmth has been associated with children’s depressive symptoms [39] and externalising behaviour problems among boys [43]. Building on these largely cross-sectional studies, we sought to examine trajectories of mental health over time for children exposed to different parenting styles.

Finally, we examined the relationship between families’ socio-economic status and the course of children’s mental health. Many studies have shown that indicators of social disadvantage (i.e., low parental income, low educational attainment, unemployment and neighbourhood disadvantage) are associated with poor child mental health [11, 12, 44]. For example, in a recent national mental health study of Australian children and adolescents aged 4–17 years old, Lawrence, Johnson (1) found that low family income, low parental education and unemployment was associated with higher rates of mental disorders in the previous 12 months. We sought to extend this previous research by examining the relationships between socio-economic status and children’s mental health beyond a single time point to examine the course of symptoms over time.

### Aims and hypotheses

In summary, the overall objective of this study was to address the lack of longitudinal research on the mental health of children as they make the transition into adolescence. The aims of this study were to explore the association between key social risk factors (child temperament, parent mental health, parenting behaviour, and socioeconomic status) and change in mental health symptoms over a 10-year period.

## Methods

### Study sample and design

The Longitudinal Study of Australian Children (the LSAC) is a national longitudinal study that commenced in 2004 [45]. Guided by a bioecological model of child development [46], data are collected every two years on child, parental, family, community and school characteristics that influence children’s development at different ages [25, 47].

The study uses a cross-sequential design of biennial face-to-face visits with the family and study child. Data from the child cohort were collected in 6 waves at 4–5, 6–7, 8–9, 10–11, 12–13, and 14–15 years of age (Table 1).

**Table 1:** Age range, sample size and study retention, Waves 1–6.

Wave	Sample size	Sample retention (%)	Age in months	
			Range	Mean
Wave 1 (2004)	4,983		51–67	57
Wave 2 (2006)	4,464	89.6	75–94	82
Wave 3 (2008)	4,331	86.9	95–119	106
Wave 4 (2010)	4,169	83.7	121–140	130
Wave 5 (2012)	3,956	79.4	146–166	155
Wave 6 (2014)	3,537	71.0	168–190	179

The LSAC sampling frame was extracted from the Medicare Australia enrolment database [48, 49]. The sample was chosen to be representative of Australian children in the selected age cohort, proportional to their regional distribution. Stratification was used to ensure proportional geographic representation, and a two-stage clustered design was used: first selecting postcodes, then children within postcodes.

The initial sample was broadly representative of the general Australian population compared with the 2001 Census, but slightly under-representative of families who were single-parent, non-English speaking and living in rental properties. These were the same characteristics associated with attrition over time [50].

### Measures

#### Children’s mental health

Children’s mental health was assessed at each wave of the LSAC using the maternal report of the SDQ [51]. The SDQ comprises 25 items that measure 5 sub-scales: emotional symptoms; conduct problems; hyperactivity/inattention; peer relationship problems; and prosocial behaviour. The questions for each sub-scale are the same at each age, other than 2 questions on the conduct disorders sub-scale which vary slightly for 4 year olds, with 2 items on antisocial behaviour replaced by items on oppositionality.

The SDQ total score is a sum of scores on 20 items (omitting prosocial items), with higher scores representing poorer psychosocial functioning. Each item is scored a 0, 1 or 2 based on the scoring key (not true, somewhat true, certainly true), giving a maximum score of 10 for each sub-scale and an SDQ total ranging from 0–40. Following the SDQ scoring guide [52], these scores have been scaled up pro-rata if at least 3 items were completed for the sub-scale. If 2 or less items are completed, the sub-scale (and hence, the total SDQ) was treated as missing or incomplete.

#### Socio-economic status (SES)

Three measures of SES were included in our model: maternal education, family income, and neighbourhood socio-economic disadvantage.

Maternal education was grouped into the following categories; Year 10 or less (26.7%), Year 11 or 12 (44.8%), and a university qualification (28.5%). Mothers with post-school qualifications that did not include a university qualification (e.g. a trade certificate) were grouped according to the highest level of schooling completed.

Families were asked to report their total weekly family income from all sources. Responses were partitioned into relatively equal categories: those families earning under $600, $600-$999, $1000-$1499, $1500-$1999, and $2000 or more per week. For analytic purposes, income was treated as a continuous variable.

The neighbourhood socio-economic index for areas (SEIFA) disadvantage index summarises information from the Australian Census of Population and Housing as this relates to economic and social disadvantage in small areas, such as low income, low educational attainment and high unemployment [53]. This data was linked at the Statistical Local Area level, or, where this was not available, at the postcode level. SEIFA scores range from 790 to 1,160, with 1,000 designed as the national average. Higher scores indicate higher levels of neighbourhood advantage.

#### Maternal psychological distress

We used the Kessler 6 (K6) to measure maternal non-specific psychological distress [54]. Mothers were asked how often in the past 4 weeks they had felt nervous; hopeless; restless or fidgety; that everything was an effort; so sad that nothing would cheer you up; or worthless, and responded on a 5-point scale from 0 = all of the time to 4 = none of the time. Responses were reverse coded, summed and adjusted to generate a total score ranging from 0–24, where higher scores represented higher levels of non-specific psychological distress. Previous research has shown that scores of 8 and above indicate probable mental illness and scores of 13 or above indicate a probable *serious* mental illness [55, 56]. At Wave 1, 16% of mothers had K6 scores of 8 or above, and 3.5% of mothers had K6 scores of 13 or above.

#### Maternal parenting

Maternal parenting behaviour was assessed by self-report using measures of parenting warmth and hostility developed for the LSAC [38]. Maternal warmth was assessed on a 6-item scale, including questions such as ‘How often do you express affection by hugging, kissing and holding this child?’. Maternal hostility was assessed on a 5-item scale, with items including questions such as ‘How often do you get annoyed with this child for saying or doing something he/she is not supposed to do?’. Responses to each item were on a 5-point Likert scale, ranging from “almost never” to “always/almost always”. Items for each measure were summed to create a composite score also scaled from 1 to 5, with higher scores representing higher levels of maternal warmth and hostility respectively.

#### Child temperament

Child temperament was measured at Wave 1 of the LSAC by administering the Short Temperament Scale for Children (STSC) [57] to the person designated as the primary caregiver. The STSC measures persistence, reactivity and sociability. Persistence refers to a child’s ability to stay focused on tasks, reactivity refers to irritability, negative mood and high-intensity negative reactions, and sociability refers to a child’s tendency to approach novel situations and people or conversely to withdraw and be wary. Each temperament dimension was assessed using 4 items, rating the frequency of the behaviours on a 6-point Likert scale of occurrence from “almost never” to “almost always”. Where data were missing for any of the items making up a dimension of temperament respondents were coded as missing for that variable.

### Data analysis

The data were analysed using multi-level models [58]. This approach makes use of repeated measurements on individuals and accounts for the correlation of within-person observations over time.

Our growth curve modelling utilised a two-level nested structure. Level 1 was the within-child model while level 2 was the between-child model. The within-child component allowed each child to have a unique mental health trajectory and the between-child component represents variation in mental health growth parameters (mean and slopes). The PROC MIXED procedure in SAS 9.4 was used to fit these models [59].

The model was parameterised using the child’s age in years minus 48 months. That is, the intercept in our model corresponds to SDQ scores at age 4. The model allows us to estimate which predictors are associated with SDQ scores at age 4, and with change in SDQ scores with each additional year of age. All predictor variables were treated as time-invariant predictors, and measured as of Wave 1 when children were 4–5 years old. Interaction terms between each predictor and age were estimated to examine differences in slopes (rate of change with each additional year of age).

All continuous variables were grand mean centred, excepting age, which was “centred” to 4 years. Thus, for a child aged 4, the intercept can be interpreted as the mean SDQ when all continuous predictors take their mean values and when categorical variables take their reference values. For categorical variables (child sex, and maternal education) a reference category was selected (female, and Year 11 or 12 education, respectively).

## Results

### Sample characteristics

The analytic sample for the final growth model comprised the 3,731 children for whom there was no missing data on the predictors at Wave 1 of the study. This group was compared to the full sample of children as at Wave 1, to determine whether an imputation strategy was required. This comparison revealed only modest differences. The mean SDQ score for the full sample was marginally higher than the analytic sample (9.4, compared with 9.2), and there were minor differences in income and education; 26.7% of the full sample had mothers with less than Year 10 education, compared with 25.2% in the analytic sample, and 17.5% of the full sample reported weekly incomes of less than $600 a week, compared with 15.1% of the analytic sample. The modal classes for income and education were unchanged. For all other predictors considered, the difference in mean score between the full sample and the analytic sample were less than one decimal place. As the analytic sample appeared to be representative of the full sample and that imputing missing data would make little substantive difference to either the magnitude or direction of the associations reported, an imputation strategy was not pursued.

### Descriptive analyses

The distribution of SDQ scores at each wave of the study, and the predictors used in the analysis are provided in Table 2.

**Table 2:** Descriptive characteristics for variables.

Variable	Range	Mean (%)	SD
Child SDQ at Wave 1	0–35	9.2	5.3
Child SDQ at Wave 2	0–34	7.7	5.0
Child SDQ at Wave 3	0–35	7.4	5.2
Child SDQ at Wave 4	0–33	7.8	5.5
Child SDQ at Wave 5	0–34	7.3	5.5
Child SDQ at Wave 6	0–35	7.0	5.4
Maternal psychological distress (K6) at Wave 1	0–24	4.1	3.7
Child sex			
	Male	51.3%	
	Female^a^	48.7%	
Family income at Wave 1			
	Under $600	15.1%	
	$600-$999	23.9%	
	$1000-$1499^a^	25.9%	
	$1500-$1999	17.9%	
	$2000 or more	17.3%	
Maternal education at Wave 1			
	Year 10 or less	25.2%	
	Year 11 or 12^a^	44.5%	
	University	30.3%	
SEIFA disadvantage index at Wave 1^b^	660–1160	1011.7	57.3
Maternal parenting warmth at Wave 1	1–5	4.4	0.5
Maternal parenting hostility at Wave 1	1–4.8	2.2	0.6
Persistent child temperament at Wave 1	4–24	15.7	3.8
Sociable child temperament at Wave 1	4–24	15.3	4.9
Reactive child temperament at Wave 1	4–24	10.7	3.7

^a^ reference category;

^b^ change in SEIFA was assessed per 50 points change

The mean child SDQ score at Wave 1 was 9.2. SDQ scores decreased to a mean level of 7.0 by Wave 6. The mean maternal K6 score at Wave 1 was 4.1, indicating a low level of distress. There was an even distribution of boys and girls in the study sample. The modal category of family income at Wave 1 was $1000–1499 a week. The modal category of maternal education at Wave 1 was a year 11 or 12 education. The average family lived in a neighbourhood with a SEIFA index score of 1011.7. Children were generally exposed to high levels of maternal warmth (4.4), low levels of maternal hostility (2.2), and had temperaments high in persistence (15.7) and sociability (15.3), and low in emotional reactivity (10.7).

The variables were screened to ensure no violations of the assumptions of multi-level models. An inspection of SDQ scores revealed a non-normal distribution. The Box-Cox transformation method was used to find the optimal transformation for SDQ scores, which for this sample was the square root of SDQ. Models were subsequently estimated using both transformed and non-transformed SDQ scores. An inspection of model results found no substantive differences between transformed and untransformed SDQ scores, and the analyses presented in this paper use untransformed SDQ scores. An examination of model residuals indicated that no transformation of the predictors was required, and that the relationship between the predictor set and child SDQ scores were adequately modelled with linear relationships. The inter-correlations between predictors were modest, and there were no concerns with multicollinearity.

### Multi-level models

The multivariate model estimating SDQ scores at age 4, and the change in scores with each additional year of age, is described in Table 3.

**Table 3:** Final growth model estimating SDQ scores.

	Model Coefficient	95% CI
**Fixed Effects**		
For INTERCEPT		
Intercept (age 4)	8.376***	8.149, 8.602
Maternal K6	0.195***	0.160, 0.230
Male (vs female)	0.930***	0.683, 1.177
Family income quintile	-0.459***	-0.564, -0.354
Maternal education University (vs Year 11 or 12)	-0.389*	-0.692, -0.087
Maternal education Year 10 or less (vs Year 11 or 12)	0.499**	0.189, 0.808
Neighbourhood-level advantage	-0.277***	-0.392, -0.162
Maternal warmth	0.047^n.s.^	-0.246, 0.340
Maternal hostility	2.324***	2.082, 2.566
Child persistence	-0.323***	-0.357, -0.288
Child sociability	-0.039**	-0.065, -0.014
Child reactivity	0.304***	0.267, 0.341
For SLOPE (interaction with child age)		
Intercept (age 4)	-0.161***	-0.192, -0.129
Maternal K6	0.007**	0.002, 0.012
Male (vs female)	0.006^n.s.^	-0.029, 0.040
Family income quintile	0.015*	0.001, 0.030
Maternal education University (vs Year 11 or 12)	-0.002^n.s.^	-0.044, 0.039
Maternal education Year 10 or less (vs Year 11 or 12)	0.015^n.s.^	-0.029, 0.059
Neighbourhood-level advantage	-0.009^n.s.^	-0.026, 0.007
Maternal warmth	0.038^n.s.^	-0.003, 0.079
Maternal hostility	-0.135***	-0.169, -0.101
Child persistence	0.007**	0.002, 0.011
Child sociability	0.009***	0.006, 0.013
Child reactivity	-0.012***	-0.017, -0.007
**Variance Components**		
*Level -1*		
Within-person (residual variance)	8.242	
*Level-2*		
In initial status (age 4)	8.636	
In rate of change	0.124	
Covariance	-0.146	
Correlation	-0.141	
Observations	19,247	
N	3,731	
Average number of observations per child	5.2	

* P < = .05;

** P < = .01;

*** P < = .001;

n.s. = not statistically significant

The intercept (8.38) is the mean SDQ for a child aged 4 years when predictions are made at the mean of all other continuous control variables and for the reference categories for categorical predictors. The effect for ‘years’ is the decrease in SDQ score (-0.16) for every year subsequent to baseline. Both of these terms were statistically significant.

Of all predictors in the multivariate model, only maternal warmth was not significantly associated with effects at baseline, with all other variables associated with SDQ scores at age 4–5 years. Maternal K6, family income, maternal hostility, child persistence, child sociability reactivity and child reactivity showed statistically significant interactions with time.

Higher maternal psychological distress was associated with higher SDQ scores at age 4, where SDQ scores were 0.20 points higher for each unit (point) increase in K6 above the mean. The interaction between initial maternal psychological distress and child age was statistically significant, with higher levels of maternal psychological distress associated with smaller decreases in SDQ scores over time.

Boys had higher average SDQ scores than girls children at age 4 (0.93 points higher). The interaction between child sex and age was not statistically significant, and the difference between boys and girls remained constant over time.

Higher family income was associated with lower SDQ scores at age 4. An increase of one income quintile above mean income quintile at Wave 1 was associated with a 0.46 point decrease in SDQ at age 4–5 years. The interaction of initial family income and child age was statistically significant, with lower levels of family income associated with smaller rates of decline in SDQ over time.

There was a clear gradient associated with maternal education, with children of university educated mothers having starting SDQ 0.39 points lower scores than children of mothers who had completed year 11 or 12. Children of mothers who had a year 10 education had starting SDQ scores 0.50 points higher than children of mothers who had completed year 11 or 12. The interaction between maternal education and years was not statistically significant.

Children living in more advantaged neighbourhoods (SEIFA) also had lower SDQ scores at age 4 years, with a decrease of 0.28 SDQ points for every 50 SEIFA points above mean SEIFA at Wave 1. The interaction between neighbourhood disadvantage and child age was not statistically significant.

Higher maternal hostility ratings were associated with higher SDQ scores at age 4 years, with an increase of 2.32 points in SDQ for every hostility point above mean hostility at Wave 1. The effect of maternal hostility at Wave 1 had a negative interaction with child age, so that the children of mothers with higher maternal hostility had SDQ scores that decreased at a faster rate over time than children of mothers with lower hostility.

Higher levels of temperamental persistence were associated with lower SDQ scores at age 4 years, with a decrease of 0.32 SDQ points for every persistence point above mean persistence at Wave 1. Persistence had a statistically significant interaction with child age, with children with higher persistence scores decreasing in SDQ over time at a lower rate than children with lower persistence scores.

The greater the child’s temperamental sociability, the lower the child’s starting SDQ scores with a decrease of 0.04 SDQ points for every sociability point above mean sociability at Wave 1. The effect of sociability at Wave 1 had an interaction with child age, so that the decrease in SDQ over time was slightly lower for children with higher sociability scores.

The higher temperamental reactivity at Wave 1 the higher the child’s SDQ score at age 4, with an increase of 0.30 SDQ points for every reactivity point above mean reactivity at Wave 1. The effect of temperamental reactivity at Wave 1 had a negative interaction with child age, so that the children with higher reactivity had SDQ scores that decreased at a faster rate over time than children with lower reactivity.

#### Interpretation

In order to compare the relative impact of the time-invariant predictors in the model on child mental health, prototypic trajectories were plotted for each predictor, using results from the fully adjusted multivariate model. For continuous predictors, trajectories were plotted for the approximate 15^th^, 50^th^, and 85^th^ percentiles in order to define low, middle and high risk groups for comparative purposes (Table 4). For categorical predictors, such as child sex, existing categories were used. This approach provided an assessment of the relative effect of different predictors on child mental health across different scales, while controlling for other predictors.

**Table 4:** Risk categories for time-invariant predictors.

Predictor	Low risk (15^th^ percentile)	Middle risk (50^th^ percentile)	High risk (85^th^ percentile)
Mother K6	0	3	7
Child sex	Female	n/a	Male
Weekly family income	$2000 or more	$1000–1499 per week	$600 or less
Maternal education	University educated	Year 11 or 12	Year 10 or less
SEIFA	1070	1000	950
Warmth	4.83	4.4	3.83
Hostility	1.5	2.0	2.75
Persistence	19	15.7	11
Sociability	9	15	21
Reactivity	6.5	10	14

**Fig 1:**
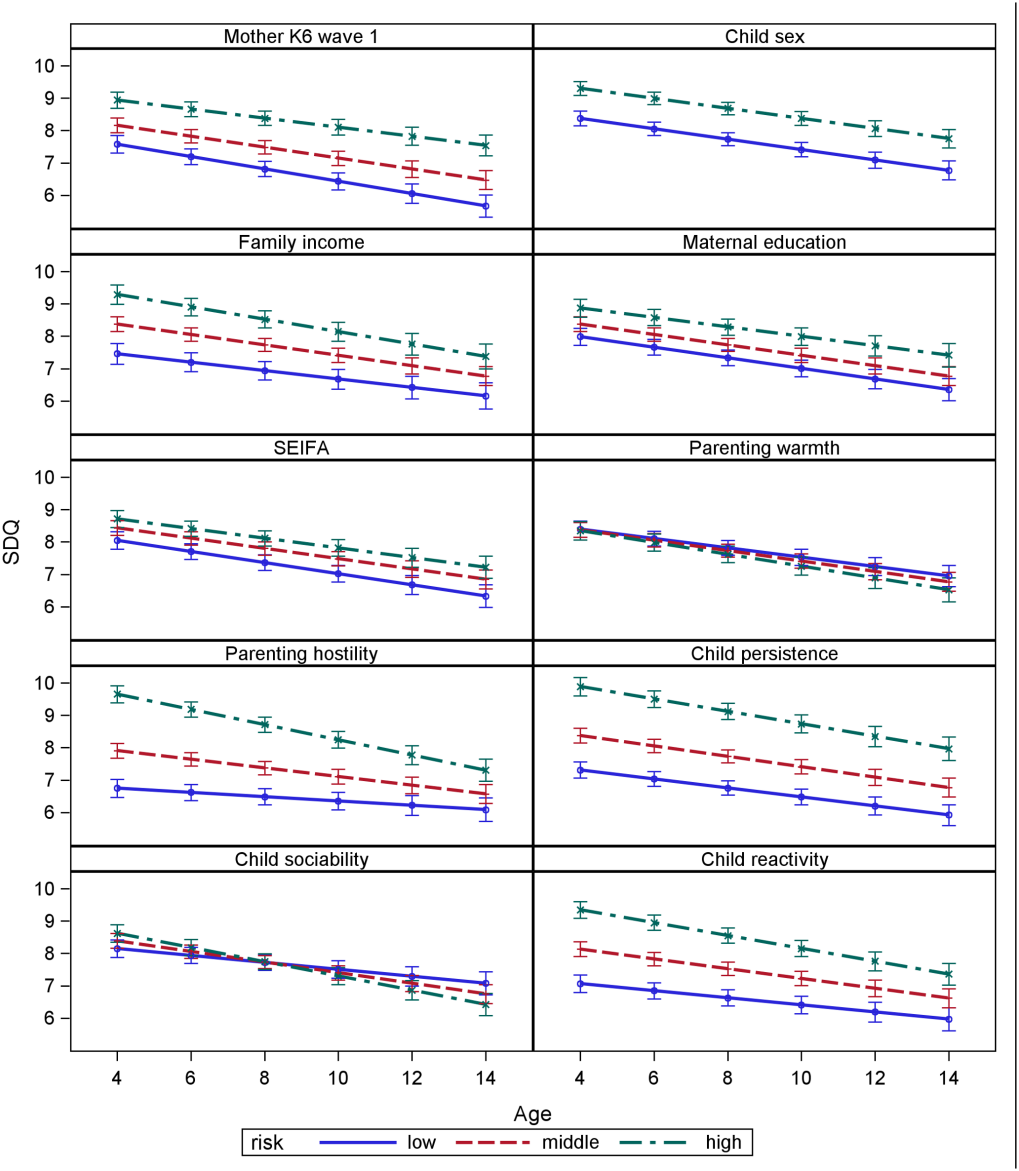
Prototypical trajectories of SDQ.

These prototypic trajectories were estimated in SAS using the ESTIMATE function in Proc Mixed [59], and have been depicted in Fig 1. This representation of the data highlights several features: 1) there is an overall downward trend in SDQ scores across all children as they get older; 2) children start at different points, based on exposure to risk factors; 3) the rate of change over time varies by risk factor exposure; and 4) gaps in SDQ based on risk factors change over time. The error bars depict the 95% confidence intervals in these estimates.

To examine the magnitude of change over time, Table 5 provides the estimated scores for each low and high group at age 4 and 14 years, along with the estimated difference scores between the high and low groups. Table 5 shows suggests that children of mothers with a K6 of 0 at Wave 1 (low) had SDQ scores 1.36 points less than the children of mothers with K6 scores of 8 at Wave 1 (high). SDQ scores decreased for children across time, but more so for the children of mothers with lower K6 scores. By age 14, the difference between ‘low’ risk’ and ‘high risk’ had increased to 1.87 SDQ points (Table 5).

**Table 5:** Projected SDQ at ages 4 and 14.

	Age 4			Age 14			
Predictor	Low risk	High risk	difference	Low risk	High risk	difference	Change in gap from age 4 to 14
Maternal K6	7.58 (7.31, 7.84)	8.94 (8.69, 9.19)	1.36 (1.12, 1.61)***	5.67 (5.33, 6.02)	7.54 (7.22, 7.87)	1.87 (1.54, 2.20)***	0.51 (0.15, 0.86)***
Child sex	8.38 (8.15, 8.6)	9.31 (9.09, 9.52)	0.93 (0.68, 1.18)***	6.77 (6.47, 7.06)	7.75 (7.47, 8.04)	0.99 (0.67, 1.31)***	0.06 (-0.29, 0.40)^n.s.^
Weekly family income	7.46 (7.14, 7.78)	9.29 (8.99, 9.59)	1.84 (1.42, 2.25)***	6.16 (5.75, 6.57)	7.38 (6.99, 7.76)	1.22 (0.67, 1.76)***	-0.62 (-1.21, -0.03)*
Maternal education	7.99 (7.72, 8.25)	8.87 (8.6, 9.15)	0.89 (0.53, 1.25)***	6.35 (6.01, 6.70)	7.42 (7.05, 7.78)	1.06 (0.59, 1.53)***	0.17 (0.33, 0.68)^n.s.^
SEIFA	8.05 (7.78, 8.32)	8.71 (8.45, 8.98)	0.66 (0.39, 0.94)***	6.33 (5.99, 6.68)	7.22 (6.88, 7.56)	0.89 (0.53, 1.25)***	0.22 (-0.17, 0.61)^n.s.^
Warmth	8.4 (8.14, 8.65)	8.35 (8.06, 8.64)	-0.05 (-0.34, 0.25)^n.s.^	6.95 (6.62, 7.28)	6.52 (6.15, 6.9)	-0.43 (-0.81, -0.05)*	-0.38 (-0.79, 0.03)^n.s.^
Hostility	6.75 (6.47, 7.03)	9.65 (9.39, 9.92)	2.90 (2.60, 3.21)***	6.09 (5.72, 6.45)	7.30 (6.96, 7.64)	1.21 (0.82, 1.61)***	-1.69 (-2.11, -1.27)***
Persistence	7.31 (7.06, 7.56)	9.89 (9.61, 10.17)	2.58 (2.31, 2.85)***	5.92 (5.6, 6.24)	7.97 (7.60, 8.34)	2.05 (1.69, 2.41)***	-0.53 (-0.92, -0.15)**
Sociability	8.15 (7.88, 8.43)	8.62 (8.35, 8.89)	0.47 (-0.17, 0.78)^n.s.^	7.08 (6.72, 7.43)	6.42 (6.08, 6.77)	-0.65 (-1.05, -0.26)**	-1.13 (-1.55, -0.71)***
Reactivity	7.07 (6.79, 7.34)	9.35 (9.09, 9.61)	2.28 (2.00, 2.56)***	5.97 (5.62, 6.32)	7.36 (7.03, 7.69)	1.39 (1.03, 1.75)***	-0.89 (-1.28, -0.50)***

* P < = .05;

** P < = .01;

*** P < = .001;

n.s. = not statistically significant

At age 4, boys had SDQ scores 0.93 points higher than girls. While both groups decreased in SDQ over time, there was no statistically significant difference in rate of change over time, and the gap between boys and girls remained unchanged by age 14.

Children from families earning $600 or less a week at Wave 1 had SDQ scores 1.84 points more than the children from families $2000 or more at age 4. SDQ scores decreased across time at a slightly greater rate for children from lower income families, and by age 14 the gap had decreased to 1.22 SDQ points.

At age 4 children of university educated mothers had SDQ scores 0.89 points lower than children of mothers with Year 10 or less educations. While both groups decreased in SDQ over time, there was no statistically significant difference in rate of change over time, and the gap based on maternal education remained unchanged by age 14.

At age 4 children from disadvantaged neighbourhoods had SDQ scores 0.66 points higher than children from more advantaged neighbourhoods. While both groups decreased in SDQ over time, there was no statistically significant difference in rate of change over time, and the gap based on neighbourhood disadvantage remained unchanged by age 14.

Maternal parenting warmth was the only predictor which showed no statistically significant association with child mental health after controlling for the other factors in the model.

Children of mothers reporting high parenting hostility had SDQ scores that were 2.90 points higher than the children from mothers low in maternal hostility at age 4. SDQ scores decreased for children over time, but more so for the children of mothers with higher levels of parenting hostility. By age 14, the difference between the children of mothers low in hostility and the children of mothers high in hostility had decreased to 1.21 SDQ points.

Children with low persistence had higher SDQ scores than high-persistence children at age 4 (2.58 points higher). SDQ scores decreased for children over time, but more so for children low in persistence. By age 14, the difference children high and low in persistence had decreased to 2.05.

At age 4, children low in sociability had SDQ scores that were 0.47 points higher than children high in sociability. Again, SDQ scores decreased for children across time, but more so for children low in sociability. The difference between children high and low in sociability had reversed by age 14, so that children low in sociability (at baseline) actually had SDQ scores that were 0.65 points higher than children with high sociability.

Children higher in reactivity had SDQ scores that were 2.28 points higher than children who were low in reactivity at age 4. SDQ scores decreased for children across time, but more so for children high in reactivity. By Wave 6, the difference children high and low in reactivity had decreased to 1.39.

Maternal hostility, child persistence and child reactivity showed the largest initial impact on child SDQ, with differences of 2.90, 2.58, and 2.28 SDQ and 2.58 points between children exposed to low and high levels of these factors, respectively. The initial difference between children exposed to high and low risk circumstances for K6 and, family income were comparable in magnitude (1.36 to 1.84 SDQ points). By age 14, the gap had narrowed to 1.22 SDQ points (income), 1.21 (hostility), 2.05 points (persistence), 1.39 points (reactivity). The gap between children with high and low maternal K6 scores had increased to 1.87 SDQ points.

## Discussion

This study sought to identify key social risk factors associated with children’s mental health symptoms at aged 4–5 years and change in symptoms over a 10-year period to 14–15 years in a large Australian population-based sample. In most Australian jurisdictions, young people aged 15 years and over are permitted to work or leave school, and this study thus covers the crucial period from when children start school to when they potentially may leave school and enter the work force or post-secondary education and training [60].

We found that the main driver of change in SDQ over time was the age of the child; SDQ scores improved (decreased) as children got older. This is a somewhat unexpected finding—recent Australian research showed that while the prevalence of mental health problems is similar among 4–11 year old males and 12–17 year males, the prevalence of mental health problems is higher among females aged 12–17 years than those aged 4–11 years [1].

Our findings also show that a range of independent factors contribute to children’s mental health at age 4 years, including child sex, maternal mental health, socio-economic status (family income, maternal education, neighbourhood disadvantage), hostile parenting, and child temperament (persistence, sociability, reactivity). These factors affect children’s mental health at a crucial period before they commence school.

Our study makes a unique contribution by examining how these factors relate to child mental health in a longitudinal context. Many of these effects remain constant over time, including child sex, maternal education and neighbourhood disadvantage, whereas others became more salient with age (maternal mental health), and others became less salient with age (family income, maternal hostility, and child persistence, sociability and reactivity).

In attempting to contextualise these effects, trajectories of mental health were compared for children exposed to ‘high risk’ and ‘low risk’ circumstances for each risk factor, based on the 15^th^ and 85^th^ percentiles of continuous predictors and existing categories of categorical predictors. This approach highlights the substantive implications of interaction with time. With the exception of child sociability, the interaction with time never completely ameliorated the initial gaps based on risk factor exposure.

This persistence of effects is a crucial finding. Differences in child mental health driven by education and neighbourhood disadvantage persist from age 4–14 unchanged. While the difference in child mental health between high income and low income families changes somewhat, the gap remains. Likewise, although the effects of hostility, persistence, and reactivity are somewhat diminished between ages 4–14, a substantial gap remains. The gap between children of mothers with low and high psychological distress increases over time.

This study is among the few to examine child and adolescent mental health in a longitudinal context. The results are broadly consistent with the other longitudinal studies of child and adolescent mental health, which also found that children’s mental health is broadly consistent over time [21–23]. Beyond this pattern, the findings advance what is known about the development and course of children’s mental health symptoms as they make the transition to adolescence in several ways. First, by demonstrating the persistence of the association between risk factors and mental health over a ten year period, it makes clear the importance of these factors. Second, the study demonstrates in a multiple regression framework that these effects make an ‘independent’ contribution to child mental health, or more simply, that each effect matters. Finally, we demonstrated the magnitude of key effects–that maternal mental health, family income, maternal hostility, and temperamental persistence have similar effects on child mental health. These findings are consistent with the bioecological model [46], and argue against a simple reduction of child mental health to a limited set of risk factors.

Our findings are consistent with the existing literature. The risks for child and adolescent mental health are reasonably well-described. Patel, Flisher (7) published a review of known risk factors for mental health in young people, enumerating a substantial range of risk factors. These include the socio-demographic factors listed above, as well as physical and biological risk factors, such as genetic predisposition towards certain forms of mental illness.

Patel, Flisher (7) also described the relationship between poverty and social disadvantage as complex and bidirectional: growing up in a disadvantaged household increases the risk of exposure to risk factors for mental health such as violence, inadequate education, and living in a neighbourhood characterised by absence of social networks. Poor mental health can also contribute to lower social status through reduced participation in education and employment, and increased health-care costs. As Hertzman and Boyce [61] argue, “Social environments and experiences get under the skin early in life in ways that affect the course of human development…”.

### Implications

Mental health matters to the child’s health, happiness, and onward economic contribution, and mental health affects not just the child, but the broader community, including the economic impacts associated with treatment and lost productivity. Heckman and Kautz [62] argue that ‘soft skills’ including personality and mental health matter as much as IQ in life outcomes. Left untreated, there is evidence to suggest that these factors lead to an intergenerational transfer of disadvantage [63].

We found that socio-economic disadvantage, maternal mental health, child temperament, and maternal hostility are associated with persistent and independent effects on child mental health. Addressing the effects of socio-economic disadvantage is complex. Children do not choose their parent’s level of income or education, the neighbourhood they are born into, the mental health or parenting skills of their mothers, or even their own temperament. However, these factors are hardly random–they represent systematic and structural inequalities. Despite the unfairness to the child, equalising these factors seems unlikely [64]. Instead, services need to be targeted in a way that addresses the social gradient, such as progressive or proportionate universalism. That is, an approach which is universal, but with a scale and intensity that is proportionate to the level of disadvantage [65].

There is also a need for increased support for parents experiencing psychological distress and other social health issues such as poverty in primary health care services, particularly as children and families make the transition from regular access to early childhood services (i.e., maternal and child health services) to formal education which has less focus on parent and family health. Schools are well placed to identify families and children who are experiencing difficulties and link them into appropriate supports for a range of health and wellbeing issues in the local community. The identification of children with potential mental health difficulties who could benefit from services and supports could be enabled with on-entry assessments when children arrive at school.

Finally, the pronounced and persistent association of hostile mother-child interactions and poor child mental health suggests a need for family and parenting support services that focus on helping parents to manage the demands of early parenting and difficult child behaviour. Examples of such supports include programs that assist parents to develop and strengthen their positive parenting skills, and to reduce drivers of family stress leading to parenting hostility (e.g. income support, relationship, or drug and alcohol services).

### Strengths and limitations

A particular strength of this paper is the use of 6 measurements of mental health on the same group of children over a 10 year period. We are able to demonstrate the independent and persistent influence of maternal mental health, child gender, family income, maternal education, neighbourhood disadvantage, maternal parenting hostility, and child persistence, sociability and reactivity, on child mental health.

The key limitations reflect the nature of the survey data. The present study is observational, and we cannot make causal inferences about our risk factors. This study also uses brief measures, and relies upon maternal report only across all the variables measured. It could be argued that more precision could be gained with more detailed and independent measures.

SDQ is a brief measure of mental health, and while it has excellent psychometric properties this study raises some concerns about how well it works when comparing children of substantially different ages. For example, Bøe and Hysing [66] question the extent to which an item relating to the frequency of tantrums is appropriate among older adolescents. This behaviour is more normative in 4–5 year olds than in 14–15 year olds, and a consequence, comparisons of total SDQ scores across time (and the age-related decrement in total SDQ) needs to be considered carefully.

While there is evidence showing moderate to high correlations between the SDQ and measures of general and specific psychopathology [67, 68], recent work emphasises the importance of using multiple informants, such as a combination of parent and teacher report. [67, 69]. The data presented in this study were obtained from mothers’ reports. The reliance on a single point-of-view may create difficulties in terms of ascertaining the reliability of the reports, as well as the biases associated with having a single respondent to all survey items.

We used a traditional multivariable regression approach to our analysis, considering each risk factor while controlling for every other risk factor. However, these risk factors do not typically occur in isolation [70–73]. While our analysis shows the average effect of each predictor across the whole population it does not address the combinations of risk factors experienced by sub-groups within the population. The mental health trajectories of children and adolescents experiencing multiple risk factors will be addressed in future work.

Finally, as with all survey data we need to consider the representativeness of the sample and the impact of survey attrition and item non-response. The initial sample was broadly representative of the general Australian population compared with the 2001 Census, but slightly under-representative of families who were single-parent, non-English speaking and living in rental properties. These were the same characteristics associated with attrition over time [50].

There were 4,983 children at Wave 1 of LSAC, and the analytic sample for the final model for the final growth model was 3,731 children. The mean SDQ at Wave 1 for the full sample (4,983 children) is 9.4. The mean SDQ for the analytic sample (3,731 children) is 9.2. There were also some modest differences in predictor variables. Given the modest magnitude of the differences between the initial Wave 1 sample and the final analytic sample, the authors determined that an imputation strategy was not required. Applying longitudinal population weights did not make any substantive differences to the analysis [74].

### Future research

The current paper considered a range of predictors of overall emotional and behavioural difficulties. There are several issues which we intend to further explore. First, the set of predictors considered in this paper was not comprehensive, nor did we consider multi-directional relationships or other aspects of the family environment that may change over time, including the father’s mental health and interparental conflict. Future work will examine the inter-relationships between child mental health, family relationships, and parent mental health. Second, the current paper only considered an overall measure of children’s mental health. An outstanding issue is the extent to which the findings in this paper can extend to sub-types of mental health. Future work will examine whether the same factors which predict total SDQ also predict clusters of symptoms such as emotional symptoms, conduct problems, hyperactivity/inattention, and peer relationship problems, and whether these sub-types of mental health follow similar trajectories to total SDQ.

The analyses in this paper reflect the contribution of a single respondent. As noted in the limitations section, recent work emphasises the importance of using multiple informants, such as a combination of parent and teacher report [67, 69]. Future work will address this concern using sensitivity analyses to compare maternal, teacher, and child self-reported measures of mental health.

Finally, the current paper did not address variability in the type or severity of symptoms over time. Future work will examine whether there are distinct groups of children within the overall sample that have different patterns of or severity of symptoms over time, and whether different predictors differentiate these groups.

### Summary and conclusions

This study illustrates the contribution of a range of factors to child mental health over a 10-year period. We identified the important and independent contribution of family SES, maternal mental health, maternal warmth and hostility, and child temperament. The independent contribution of these factors to child mental health suggests that multi-faceted approaches to improving child and parent mental health during the formative primary school years are needed.
